# Reduction of ureteral stent encrustation by modulating the urine pH and inhibiting the crystal film with a new oral composition: a multicenter, placebo controlled, double blind, randomized clinical trial

**DOI:** 10.1186/s12894-020-00633-2

**Published:** 2020-06-05

**Authors:** Carlos Torrecilla, Jaime Fernández-Concha, José R. Cansino, Juan A. Mainez, José H. Amón, Simbad Costas, Oriol Angerri, Esteban Emiliani, Miguel A. Arrabal Martín, Miguel A. Arrabal Polo, Ana García, Manuel C. Reina, Juan F. Sánchez, Alberto Budía, Daniel Pérez-Fentes, Félix Grases, Antonia Costa-Bauzá, Jordi Cuñé

**Affiliations:** 1 Bellvitge University Hospital, Barcelona, Spain; 2 La Paz University Hospital, Madrid, Spain; 3 Rio Hortega University Hospital, Valladolid, Spain; 4 Mateu Orfila Hospital, Maó, Spain; 5 Puigvert Foundation, Barcelona, Spain; 6 San Cecilio University Hospital, Granada, Spain; 7 Virgen de Valme University Hospital, Sevilla, Spain; 8 Álvaro Cunqueiro Hospital, Vigo, Spain; 9 University and Polytechnic La Fe Hospital, Valencia, Spain; 10 University Hospital Complex of Santiago de Compostela, Santiago de Compostela, Spain; 11 Laboratory of Renal Lithiasis Research, University Institute of Health Sciences Research (IUNICS- IDISBA), University of Balearic Islands (UIB), Palma de Mallorca, Spain; 12 Devicare S.L., Cerdanyola del Vallès, Spain

**Keywords:** Double J stent, Encrustation, Nutraceutical, L-methionine, Phytin, pH

## Abstract

**Background:**

Encrustation of ureteral double J stents is a common complication that may affect its removal. The aim of the proposed study is to evaluate the efficacy and safety of a new oral composition to prevent double J stent encrustation in indwelling times up to 8 weeks.

**Methods:**

A double-blinded, multicenter, placebo-controlled trial was conducted with 105 patients with indwelling double J stents enrolled across 9 public hospitals in Spain. The patients were randomly assigned (1:1) into intervention (53 patients) or placebo (52 patients) groups for 3 to 8 weeks and both groups self-monitored daily their morning urine pH levels. The primary outcome of analysis was the degree of stent ends encrustation, defined by a 4-point score (0 – none; 3 – global encrustation) using macroscopic and electron microscopy analysis of crystals, after 3 to 8-w indwelling period. Score was exponentially transformed according to calcium levels. Secondary endpoints included urine pH decrease, stent removal, and incidence of adverse events.

**Results:**

The intervention group benefits from a lower global encrustation rate of stent ends than placebo group (1% vs 8.2%; *p* < 0.018). Mean encrustation score was 85.12 (274.5) in the placebo group and 18.91 (102.27) in the intervention group (*p* < 0.025). Considering the secondary end points, treated patients reported greater urine pH decreases (*p* = 0.002). No differences in the incidence of adverse events were identified between the groups.

**Conclusions:**

Our data suggest that the use of this new oral composition is beneficial in the context of ureteral double J indwelling by decreasing mean, as well as global encrustation.

**Trial registration:**

This trial was registered at www.clinicaltrials.gov under the name “Combined Use of a Medical Device and a Dietary Complement in Patient Urinary pH Control in Patients With an Implanted Double J Stent” with date 2nd November 2017, code NCT03343275, and URL.

## Background

Double-J ureteral stents [1] are one of the most common indwelling ureteral devices used for treatment of obstructive uropathy, postoperative of ureteropyelic stenosis and renal transplantation [2, 3]. Their effectiveness for renal collecting system drainage has been proven [4] and their characteristic design, with both renal and vesical J-shaped curl ends, prevents stent migration [1]. However, double J ureteral stents have also been related to patient discomfort, pain, urinary tract infection and encrustation [4, 5].

A prolonged indwell time of stents, as well as a history of nephrolithiasis and urinary infections may result in encrustation of ureteral stents, and will lead to the use of endourological techniques, extracorporeal lithotripsy or open surgery to resolve these conditions [6–8].

Film-formation is a multistep process; shortly after the stent insertion, different organic molecules adhere to its surface forming conditioning film [9, 10] and the presence of bacteria attached to the stent surface was considered essential for the formation of struvite and hydroxyapatite crystals [4, 11]. Nonetheless, recent studies have demonstrated that the presence of bacteria is not compulsory and conditioning film, together with urine pH, might play a bigger role in Ca and Mg phosphate precipitates forming hydroxyapatite and brushite crystals, which result in stent encrustation [12]. Oher factors, such as urine pH and supersaturation, play an important role and several studies have shown that higher urine pH values are found in blocker patients (those in which stent obstruction is observed) compared to non-blocker ones [10, 13, 14]. Thus, stent encrustation could be minimized if urine composition is altered by reducing the urine alkalinisation and increasing the urine excretion of crystallization inhibitors.

The oral composition studied contains both urine acidifier and crystallization inhibitors, such us L-methionine (an essential amino acid recommended by the EAU Guidelines on Urolithiasis with acidifier properties for the treatment of infectious stones) and phytin (a phytate salt with demonstrated inhibitory properties of calcium stones) as active components. L-methionine directly reduces/acidifies urine pH [15, 16], whereas crystallization inhibitors [17] decrease the risk of renal stone formation [18, 19].

On the other hand, the pH meter is a medical device, which has been validated with patients [20, 21], designed for urine pH self-monitoring, enabling patients to easily control urine pH on their own and its applicability may be extended to other urological pathologies where urinary pH plays an important role, such as acid-base imbalance diseases, urinary tract infections, cystitis, painful bladder syndrome or stent encrustation [22–24].

The main goal of this study was to assess the potential in preventing double-J stent encrustation of a new oral composition in a study with indwelling times between 3 and 8 weeks, as well its efficacy and safety. Secondary objectives included urine pH decrease, stent removal, incidence of adverse events, patient’s compliance and physician’s and patient’s satisfaction. The promising data obtained pave the way to further investigations for the use of the oral composition in preventing stent complications.

## Methods

### Study design

A prospective, parallel, double-blinded, randomized and placebo-controlled trial was conducted between 9th January 2018 and 9th July 2018 at 9 public hospitals in Spain, in accordance with the Declaration of Helsinki, ethical standards, current legislation and GCPs. The study was approved by local Ethics Committees, and informed consent was obtained from all patients prior to their enrolment in the study. This study adheres to CONSORT guidelines.

### Subjects

**Fig. 1:**
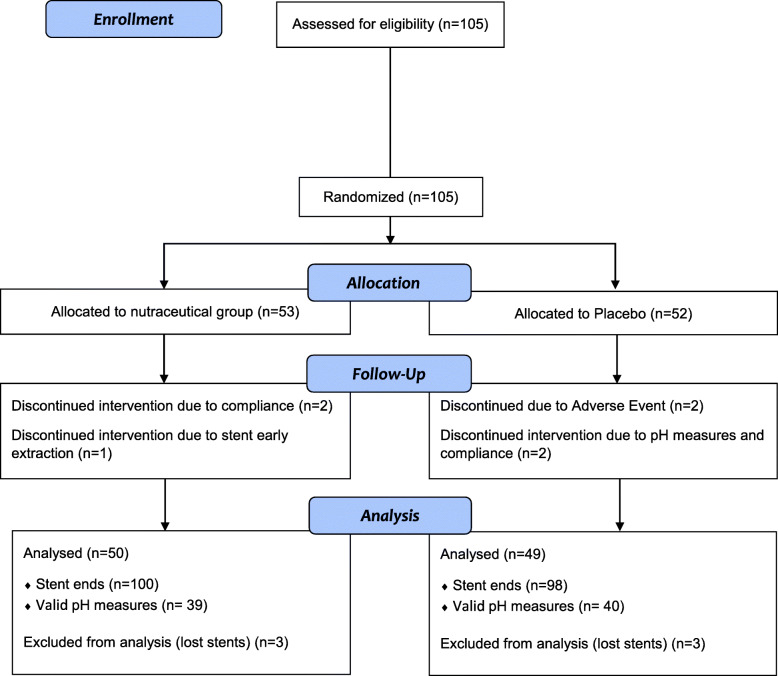
Patient flow chart and allocation

The recruitment period was from January to July 2018. Inclusion criteria comprised patients aged 18 and older, capable of daily self-monitor their urine pH, who were willing to participate and had recently implanted a double J stent (less than a week ago) or programmed for it, with an expected indwelling time below 8 weeks, maximum period of time allowed for stent indwelling according to the Ethics Committee. Exclusion criteria comprised patients with programmed stent removal prior to 3 weeks from the inclusion visit, pathologies incompatible with the consumption of the oral composition, and uric or cystinuric patients in which different pH control recommendations are needed (Fig. 1). All patients finally enrolled were stone-formers with an indwelled double J stent for urine derivation due to endourological procedures.

### Locations of data collection

The study was conducted in the following nine [9] public hospitals in Spain: 1) Hospital Universitari de Bellvitge, Barcelona; 2) Hospital Universitario La Paz, Madrid; 3) Hospital Universitario Rio Hortega, Valladolid; 4) Fundació Puigvert, Barcelona; 5) Hospital Universitario Clínico San Cecilio, Granada; 6) Hospital Universitario Virgen de Valme, Sevilla; 7) Hospital Álvaro Cunqueiro, Vigo; 8) Hospital Universitario y Politécnico La Fe, Valencia; 9) Complejo Hospitalario Universitario de Santiago de Compostela (CHUS), Santiago de Compostela.

### Treatment description

Subjects were randomly assigned in a 1:1 ratio to receive an oral composition (containing a urine acidifier and crystallization inhibitors) or placebo as investigators included them in a password-protected computer database with a pre-programmed randomization list with blocks of 2 to 4. The CRO (BioClever, Barcelona, Spain) generated the random allocation sequence, the hospitals enrolled the participants and the investigators assigned the participants to interventions. The oral composition arm consisted in oral administration of three capsules per day (1-1-1) to maintain the urine pH under 6.2, a preventive pH value, and increase the urine excretion of crystallization inhibitors to avoid stent encrustation [15, 25]. Patients in placebo arm received a treatment consisting in oral capsules with the same organoleptic and posology characteristics as the investigational compound. Both arms used a portable medical device (Lit-Control® pH Meter) to self-monitor their urinary pH every morning, and identical hygienic-dietary indications for stent care were given to all participants.

### Follow-up evaluation

Intervention and pH self-control duration ranged from 3 to 8 weeks depending on the time-lapses between the baseline visit and the stent removal. Once removed, the process consisted in submerging the stent ends in thymol to cleanse, gently letting them air dry over paper. This procedure prevents the growth of microorganisms and the crystallization progression to guarantee the correct encrustation evaluation. All analyses were carried out in a central laboratory (Laboratory of Investigation in Renal Lithiasis, Universidad de las Islas Baleares-IUNICS). Stents ends were cut and processed to examine the renal and vesical ends separately, in a homogenous fashion among the distinct enrolled centres according to protocol instructions.

### Outcome measures

#### Primary outcome

**Fig. 2:**
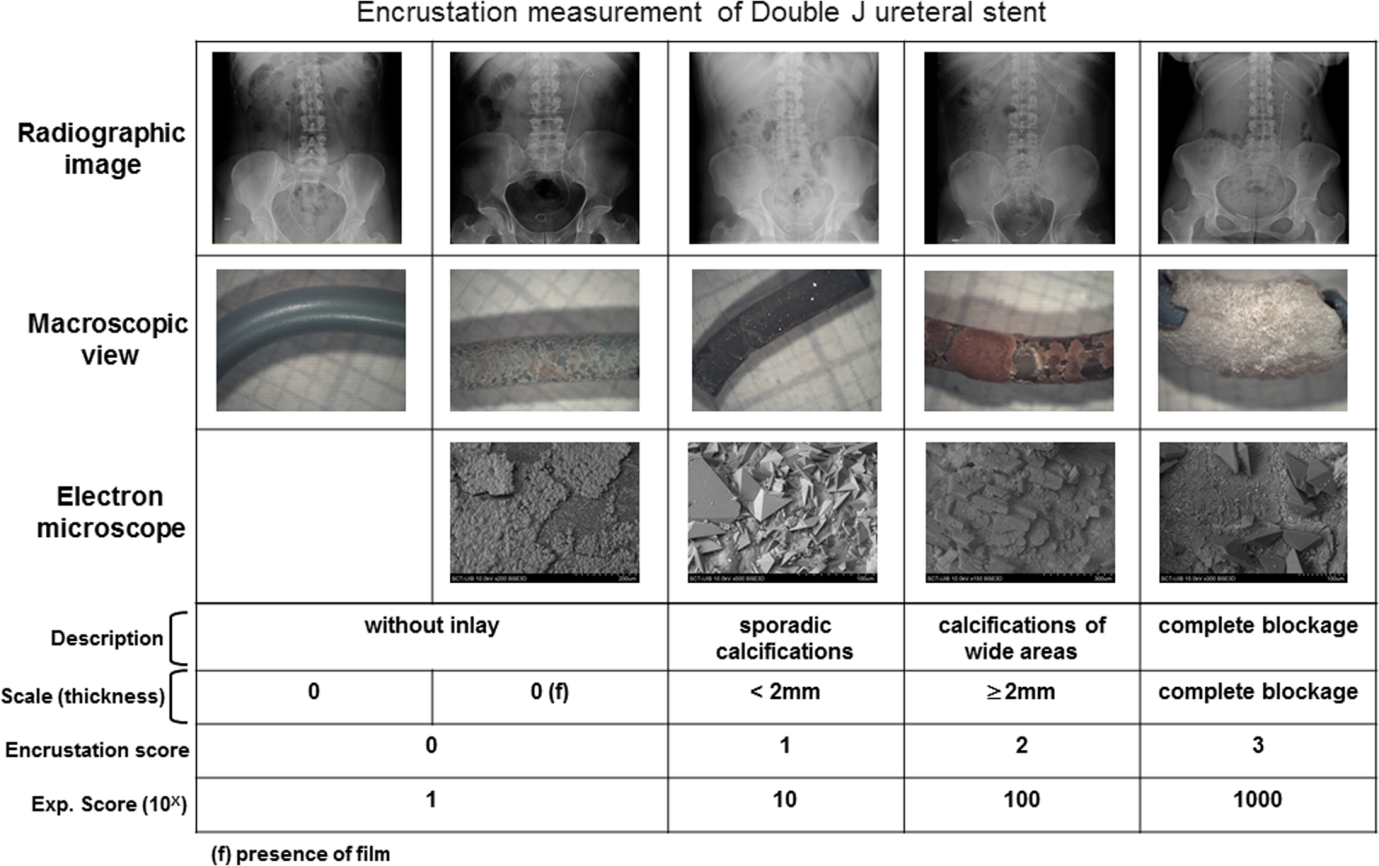
Encrustation measurement from 0 (nothing) to 4 (global encrustation) measured by radiographic image, microscopic view and electron microscope of the stent

The presence and degree of stent encrustation. A 4-level score was employed to determine the degree of encrustation based on surface and thickness (mm), (0: without inlay; 1: sporadic calcifications, < 2 mm; 2: calcification of wide areas, ≥ 2 mm; 3: global encrustation (=complete block) (Fig. 2). The 0 score was divided in 2 categories: with or without the presence of conditioning film; this division does not affect the final value of encrustation. An exponential transformation of the score was additionally applied because calcium concentration ratios, measured by Arsenazo III spectrophotometry, were following log scale. A dichotomous variable for global encrustation (score 3) was created and used in the stent ends database. The type and size of crystals were assessed by SEM and micro-analysis by dispersive energy of X-Ray, and the degree of global encrustation of each end was measured using ICP-AES spectroscopy (Fig. 2). The type of deposit, presence of bacteria, and the size and nature of the crystals were identified using scanning electron microscopy (Hitachi S 3400 N), coupled to a microanalysis by X-Ray dispersive energy (Bruker AXS GmbH, Karlsruhe, Germany).

#### Secondary outcomes

Urinary pH reduction together with duration and method of the stent extraction intervention were recorded as secondary outcomes. First morning void as a spot urine sample, was performed daily. Specifically for the quantification of urinary pH change during the study period, the following was registered: i) an hospital measure of urine pH was considered as day 0; ii) mean domiciliary values of urine pH from days 1–3 were considered the baseline of pH self-monitoring data; iii) pH domiciliary values at day 21 and iv) mean domiciliary pH values from day 4 to the end of indwelling period (21 to 56 days). The baseline was compared to pH at day 21 and to the mean pH values for the total indwelling period. Duration and method of the stent extraction intervention data were also recorded as secondary outcomes. Risk factors for encrustation development (days with implanted stent and number of previous implantations), stent-related symptoms, previous uropathies and sociodemographic data were also recorded to be studied as factors or covariates. Compliance was measured by counting returned medication, and consumption of more than 80% of the capsules was considered good adherence.

### Statistical analysis

A sample size of 47 evaluable patients per treatment group would provide approximately 80% power to detect a reduction with an effect size of 0.6 in the encrustation score in either intervention group versus placebo using a Mann-Whitney U test. The sample was increased to 105 participants considering a 10% of dropouts. Demographic and baseline characteristics and safety and tolerability data were summarised using descriptive statistics. The primary endpoint, the difference in the encrustation score between groups, was assessed using a Fisher exact test for the categorical variable global encrustation and a Mann-Whitney U test for the encrustation scores, which were also analysed using Generalized Linear Models for a Tweedie distribution with a logarithmic link, including treatment, sex, baseline pH < 6 and indwelling duration > 39 days as fixed factors and age as a covariate. Mean differences and 95% confidence limits were calculated for all comparisons between groups. Global encrustation was analysed using a logistic regression model that included treatment, sex, baseline pH < 6, first implantation, indwelling duration and age. Secondary end points as pH reduction, intervention time for stent removal or patient satisfaction were analyzed using one-way analysis of variance. Statistics for all tables, figures, and graphs were calculated from the total number of valid cases. All statistical analyses were performed on the intention-to-treat population using SPSS 22.0 software for Windows.

## Results

A total of 105 patients with a mean (SD) age of 51.6 (13.1) years were analysed (Fig. 1 and Table 1), with 198 stent ends collected from 99 subjects who wore them for an average time of 37.54 ± 13.9 days. Placebo and intervention group were comparable at baseline (*see* detailed parameters at Table 1). Concerning the presence or not of global encrustation as primary outcome, eight stent ends (8.2%) showed global encrustation in the placebo group and 1 (1.0%) in the intervention group (R.R.: 8.2 [1.04–64.06]; *p* = 0.018) in a period of 3–8 weeks, obtaining the same results than other authors in three previous studies [26–29]. The encrustation degree scores by stent end are detailed in Table 2; the analysis of all the double J stent ends resulted in encrustation levels of 85.12 (274.5) in the placebo group, and of 18.91 (102.27) in the intervention group (*p* = 0.025); difference (95% IC) is 66.21 (8.37, 124.06). These results demonstrate an 8-fold reduction in global encrustation for the experimental group, together with a striking reduction in the degree of such encrustation in every analysis, when considering distinct stent ends or the sum of all ends.

**Table 1:** Characteristics of the study population

	Placebo group		Nutraceutical group		Total		***p***-value
	N (%)	Mean (SD)	N (%)	Mean (SD)	N (%)	Mean (SD)	
Sex							
Male	28 (53.8)		30 (56.6)		58 (55.2)		0.85
Female	24 (46.2)		23 (43.4)		47 (44.8)		
Total	52 (100)		53 (100)		105 (100)		
Age		51.5 (13.2)		51.7 (13.0)		51.6 (13.1)	0.95
Previous obstructive uropathy	19 (36.5)		21 (39.6)		40 (38.1)		0.84
Previous stenting	19 (36.5)		22 (41.5)		41 (39)		0.69
Urolithiasis as cause of current implantation	41 (78.8)		41 (77.4)		82 (78.1)		0.85
Type of calculi							
Calcium oxalate	19 (46.3)		21 (51.2)		40 (48.8)		0.80
Others	22 (53.7)		20 (48.7)		42 (51.2)		
Total	41 (100)		41 (100)		82 (100)		
Stent material							
Polyurethane	23 (44.3)		20 (37.7)		43 (40.9)		0.45
Silicone	1 (1.9)		0 (0)		1 (1)		
Percuflex	28 (53.8)		33 (62.3)		61 (58.1)		
Implantation period (days)		39.7 (14.9)		35.4 (12.7)		37.54 (13.9)	0.12
Basal urinary pH	43 (100)	6.2 (0.6)	44 (100)	6.3 (0.8)	87 (100)	6.3 (0.7)	0.62

*SD* standard deviation

Group homogeneity at baseline

**Table 2:** Between groups analysis

	Placebo		Nutraceutical		Inference	
	Mean (SD)	n	Mean (SD)	n	Difference/OR (95% CI)	p
**Encrustation**						
Kidney stent end	64.73 (241.74)	49	7.66 (23.69)	50	57.07 (−11.1, 125.25)	0.89
Bladder stent end	105.51 (304.99)	49	30.16 (142.52)	50	75.35 (−19.3, 170.00)	0.65
Sum of stent ends	170.24 (513.58)	49	37.82 (159.24)	50	75.35 (−19.28, 169.99)	0.65
Maximum of stent ends	105.69 (304.93)	49	30.34 (142.49)	50	132.43 (−18.62, 283.47)	0.67
**Encrustation adjusted for baseline urine pH**						
Sum of stent ends	78.34 (158.44)	39	11.11 (32.97)	37	67.23 (19.18, 115.29)	0.006
**Encrustation adjusted for baseline urine pH, age, gender and indwelling duration**						
Sum of stent ends	57.57 (122.09)	39	18.27 (48.42)	37	39.0 (2.02, 76.57)	0.039
**Urine pH**						
pH reduction baseline (24 h) to day 21	0.39 (0.7)	28	0.86 (0.78)	32	−0.47 (−0.85, −0.084)	0.018
pH reduction days 1–3 to day 21	0.17 (0.49)	36	0.54 (0.58)	36	−0.37 (−0.62, −0.11)	0.005
pH slope	−.0061 (.013)	40	−.014 (0.02)	39	0.008 (0.00006, 0.016)	0.042
**Stent removal**						
Removal surgery time (min)	13.8 (30.5)	52	7.23 (13.5)	52		0.76
Removal surgery time (adjusted, min)	40.9 (5.8)	52	9.5 (4.15)	52		< 0.001
	**N**	**%**	**n**	**%**	**Odds ratio**	
Stent removed at first attempt	47	48.5	50	51.5	2.66 [0.49–14.37]	0.44

As for secondary outcome, the reduction in urinary pH from the baseline or day 1 to values obtained after all the indwelling period was significantly greater in the intervention group (Table 2). These data show that the administration of the new oral composition is effective in decreasing the urinary pH as a preventive measure for stent calcifications.

**Fig. 3:**
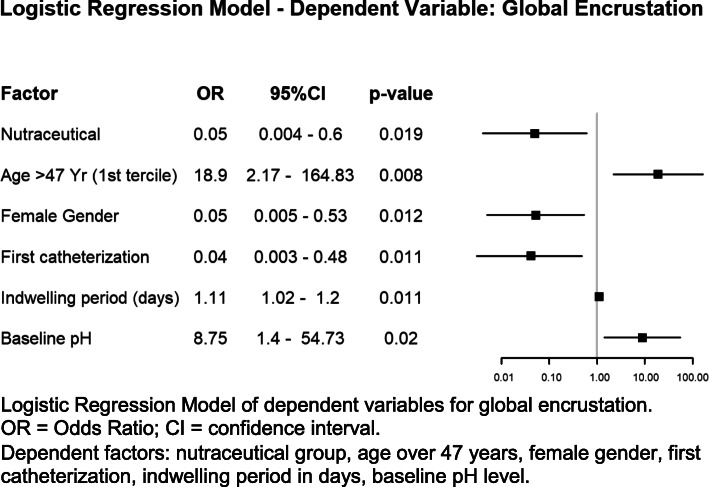
Multivariate model of Double J ureteral stent encrustation

Binary logistic regression model of all stent ends global encrustation showed a OR in the placebo group of 20.61 [95% IC: 1.66 –* 256,2; *p* = 0.019] emerging as protective factors age > 47, first implantation and baseline pH < 6 and favoring encrustation would be male gender (Fig. 3). Four (22%) of 18 patients whose mean pH level during indwelling was greater than 6 showed global encrustation in 7 stent ends and 1 (1.7%) of 58 patients with lower pH levels showed this outcome in 1 stent end (RR: 12.9 [1.4–296.7]; *p* < 0.012).

Spearman correlation between indwelling time in days and encrustation score was *ρ* = 0.212 (*p* < 0.036) for the kidney end and *ρ* = 0.153 (*p* < 0.13) for the bladder end. When separated by study group, r^2^ of encrustation score at kidney end by indwelling time was 0.079 for the placebo group and 0.018 for the nutraceutical group.

The total amount of calcium deposited in stents with encrustation scores of 3 was thousand times greater than the amount in stents with score 1 (Table 3), justifying an exponential transformation of the score. Table 4 summarize the types of scale and the magnitude of the deposits; in the 28.3% of the stents no deposit was observed in the bladder part, while in the renal part there were no deposits in 41.4%.

**Table 3:** Amount of calcium deposited on the stent

Magnitude of the scale	Id	Calcium deposit
Type 1	49 bladder	0,95 nmol / cm
	59 bladder	1,21 nmol / cm
	43 renal	0,84 nmol / cm
	49 renal	0,42 nmol / cm
	50 renal	0,85 nmol / cm
Type 3	41 bladder	330 nmol / cm
	3 bladder	346 nmol / cm
	72 bladder	244 nmol / cm
	3 renal	329 nmol / cm

**Table 4:** Characterization for stents encrustation Bladder (*N* = 99) and Renal (N = 99) ends

Percentage (%) of encrustation in stents								
	Bladder end				Renal end			
	0	1	2	3	0	1	2	3
no deposit	28.3				41.4			
OM	12.1				8.1			
COM		24.2				27.3		
COM + COD		14.1				11.1		
BRU		2.0		1.0		2.0		
HAP		1.0	1.1			1.0		
HAP + BRU		1.0		3.0				3.0
UA			3.0				2.0	
UA + COM		1.0				1.0		
bacteria		4.0				2.0		
HAP + PAM				2.0			1.0	
BRU + COD		1.0			–	–	–	–
AU		1.0			–	–	–	–

*OM* organic matter; *COM* calcium oxalate monohydrate; *COD* calcium oxalate dihydrate; *BRU* brushite; *HAP* hydroxyapatite; *UA* uric acid; *PAM* ammonium magnesium phosphate; *AU* ammonium urate

**Fig. 4:**
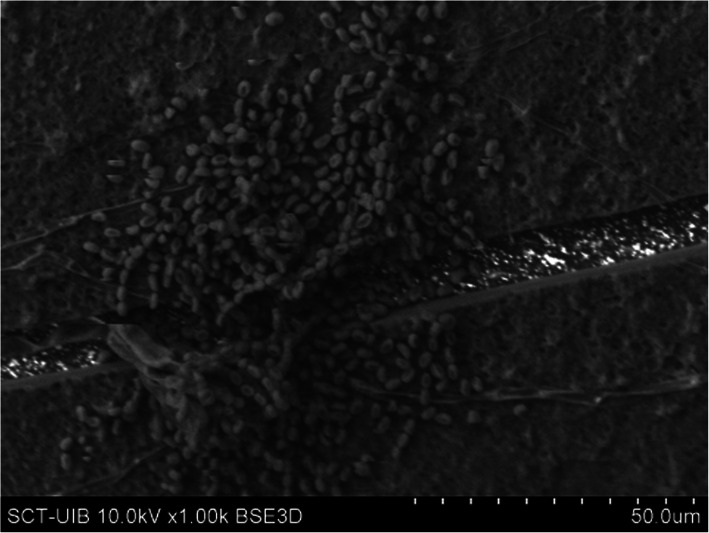
Surface of a stent covered by an organic matter layer (conditioning film) in which colonies of bacteria have developed (encrustation classified as 1)

The deposits consist mainly of organic matter only (12.1% bladder part - 8.1% renal part) or small crystals of calcium oxalate monohydrate (COM or COM + COD) developed on top of a layer of organic matter. In addition, bacteria were on the surface of the bladder part in 4.0% of the stents and on the renal part in 2.0% of the stents. In all cases, bacteria were on top of the layer of initially deposited organic matter (Fig. 4).

**Fig. 5:**
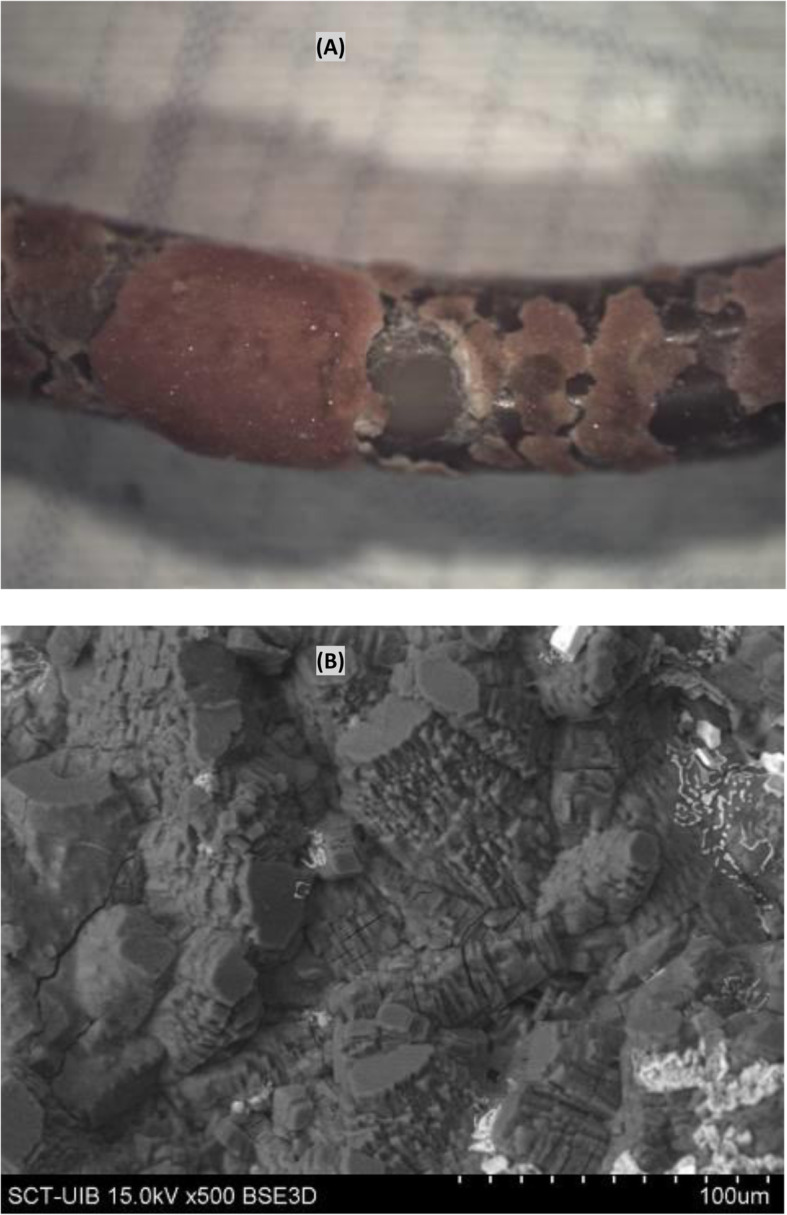
Surface of a stent covered by dihydrate uric acid deposits, classified as 2. (A) Optical image, (B) Scanning electron microscopy image

**Fig. 6:**
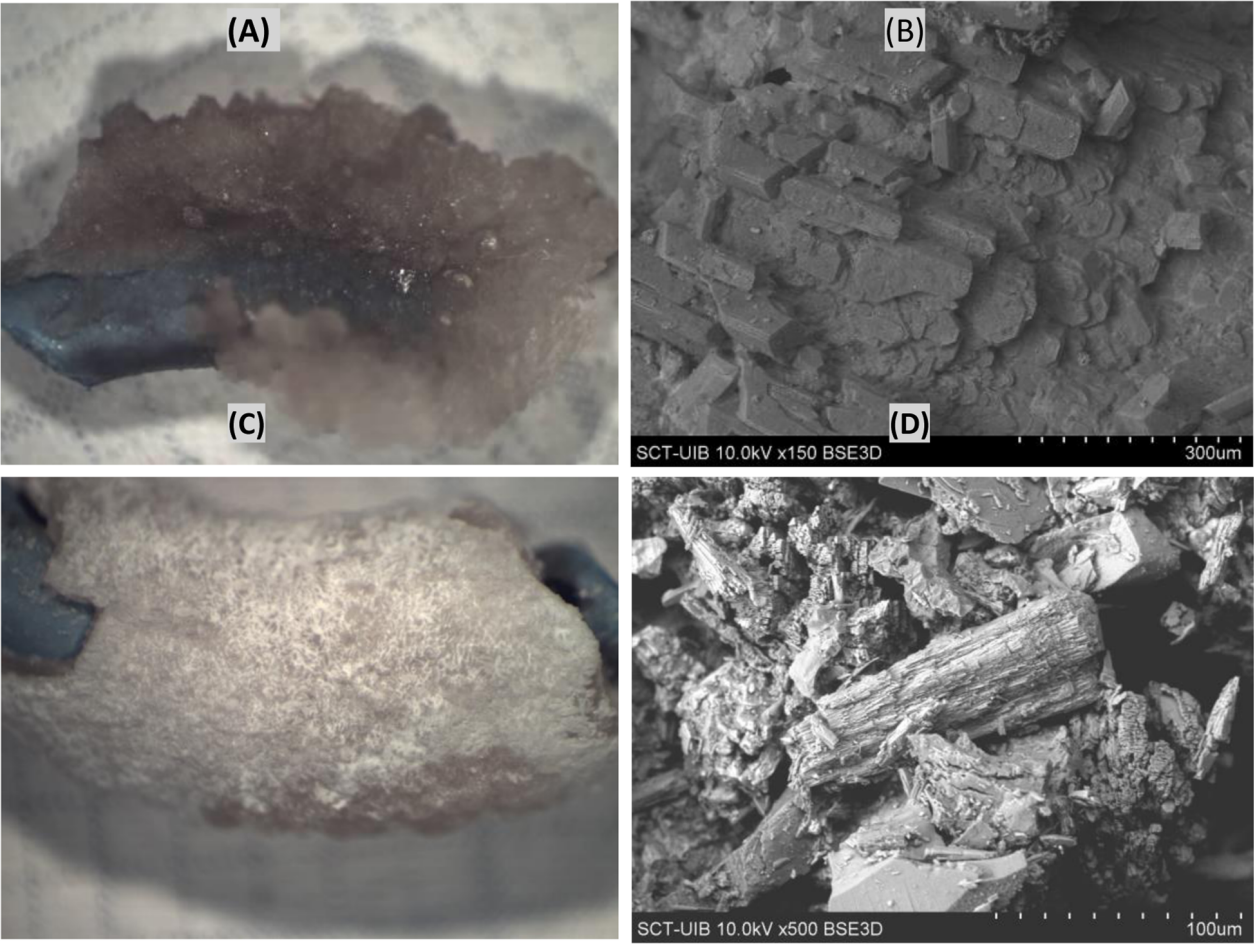
Surface of a stent covered by ammonium magnesium phosphate + hydroxyapatite deposits (A) Optical image, (B) Scanning electron microscopy image. Surface of a stent covered by brushite + hydroxyapatite deposits (C) Optical image, (B) Scanning electron microscopy image

The non-continuous deposits of thickness greater than 1 to 2 mm, mainly consisted of hydroxyapatite (1.1% in the bladder part), hydroxyapatite+ ammonium magnesium phosphate (1.0% in the renal part) and uric acid (3.0% in the bladder and 2.0% in the renal part, Fig. 5). Larger depositions, which can cause obstructions and/or complete block, were mainly brushite and hydroxyapatite (3.0% in the renal part and 4.0% in the bladder part, shown in Fig. 5), and magnesium ammonium phosphate (2.0% in the bladder part, Fig. 6). Although the deposits of magnesium ammonium phosphate are clearly of bacterial colonization origin, no bacteria were detected in the crystals.

Fifteen patients (37.5%) in the placebo group and 12 (30%) in the intervention group took less than 80% of prescribed doses (*p* = 0.6). Twelve patients (11.4%) in the placebo group and 14 (13.3%) in the intervention group failed to provide valid pH measures due to inadequate use of the device.

Three patients in the placebo group reported mild adverse events (2 nausea and 1 hot flashes) and 3 in the intervention group (1 diarrhea, 1 blurry vision and 1 dyspepsia). Two patients in the placebo group discontinued the study due to adverse events. No additional measures needed to be taken for the rest of the patients due to adverse effects. Six patients in the placebo group and 6 patients in the intervention group were prescribed with antibiotics due to positive baseline urine cultures.

## Discussion

The calcification phenomenon has relevant clinical consequences that may compromise stent removal. When indwelling time increases, encrustation prevalence increases proportionally [7, 26, 29, 30] and global encrustation can occur, leading to the use of endourological techniques, extracorporeal lithotripsy or open surgery to resolve these conditions [8, 31]. Although heavily encrusted stents clearly do pose significant problems, minor encrustations can also challenge the endourologist, particularly if occurring frequently and repetitively [27]. Some publications indicate that the mere presence of a biofilm in the stent increase patient’s discomfort and lower urinary tract symptoms (LUTS) [11, 32], which may increase inflammation, tissue damage and eventually affect stent removal. To this date, no oral treatment to prevent or decrease stent encrustation have been proposed.

The degree of stent encrustation was strikingly reduced in the experimental group treated with the oral composition, when considering each stent end separated or their sum, as well when adjusting the data for baseline urine pH, age, sex, previous implantation and indwelling duration. Particularly for those stents with a global encrustation value, the difference between the intervention group and placebo yielded a relative risk of 8.2 and this effect was enhanced by baseline pH level.

The microscopic study of the stents indicated that organic matter in the urine (macromolecules or cellular debris) is first deposited on the stent forming a layer (conditioning film) that is several micrometers thick (Fig. 2, encrustation definition 0(f)). The thickness and composition of the conditioning film depend on the urine composition of the patient.

For patients with non-lithogenic urine (no hypercalciuria, no hyperoxaluria, no hypocitraturia, and a urinary pH between 5.5 and 6.2) and no bacterial colonization of the urine, organic matter deposits can occur, and act as heterogeneous nucleants that support the growth of COM crystals over 2 to 3 months [33, 34]. This growth is very slow, forming only a thin layer (thickness of several micrometers) (Fig. 2). The underlying mechanism may be analogous to the formation of COM stones in renal cavities [33]. If a patient has a high level of urinary calcium, then COD crystals may develop.

If bacteria are present, they can colonize the stent surface and grow while embedded in the initially deposited organic matrix (Fig. 4). The biofilm resulting from infection by urease-producing bacteria increases the urinary pH and leads to the formation of carboxyapatite and magnesium ammonium phosphate crystals (Fig. 6). Depending on bacterial activity, these crystals can range from small deposits to large concretions, and, in many cases, they obstruct the inflow and outflow through the stent, and make the stent extraction much more difficult for the urologist. The most common bacteria in these deposits is *P. mirabilis* [35, 36]. It is interesting to observe how the presence of bacteria on the organic matter layer has been detected, forming the biofilm, but they have not been identified on the magnesium ammonium phosphate crystals, which are clearly infectious. This can be explained considering that the bacteria are installed in the areas between the organic matter and the surface of the crystalline deposit, thus being also protected from the action of antibiotics.

For urine with a pH higher than 6.2 and no bacterial colonization, significant deposits of calcium phosphate can develop depending on the specific conditions. In particular, when the urine has a high calcium concentration, a citrate deficit, and a pH greater than 6.2, large deposits of brushite can build (Fig. 6) [33, 34]. Under these conditions, large COD crystals can also occur. When the calcium and magnesium concentrations are low, large hydroxyapatite deposits can develop. For urine with a pH less than 5.5, major deposits of uric acid can develop (Fig. 5). It is important to point out that, in urinary pH values between 5.5 and 6.2, the crystalline development occurs at such a rate that does not allow the development of large deposits and consequent obstructions.

The multivariate models showed that the formation of deposits in the double J stent ends is a multifactorial process dependent on patient’s previous implantation, duration of the implantation period, baseline pH level, and the use of an oral composition (Fig. 3). Both oral composition and baseline pH are independent factors that prevent stent encrustation. A mean pH greater than 6.2 during indwelling time increased 12.9 times the risk of global encrustation of a stent end. In addition, the experimental group has a higher urinary pH decrease from baseline to the end of the indwelling period. The fact that the oral composition studied consists in an acidifier (L-methionine) plus an inhibitor (phytin) may account for it, since both components have a synergic effect on reducing urine pH and inhibiting urine crystallization, respectively, which may prevent encrustation [12, 18, 19].

A better adherence to treatment could add more value to the final data; 37.5% of patients in the placebo group and 30% in the experimental took less than 80% of prescribed doses. Additionally, both intervention and placebo groups lowered their urine pH levels; this may be since hygienic-dietary indications for stent care were given to all participants and to the daily urine pH self-monitoring carried out by both groups. Patients scored their satisfaction with the pH meter with an average of 8 over a 0 to 10 scale.

This study has some limitations. It would be useful if metabolic urine studies were performed prior and after the administration of the oral composition and/or the placebo. However, most of the cases included in our trial came from the emergency room (ER) or from peri-surgical situations, making difficult to collect urine samples for metabolic analysis. In addition, it was considered a possibility of bias in the urinary metabolic parameters due to such hospitalization and surgical interventions. It is a pioneer study, consisting in the first controlled, prospective, randomized and multicenter trial collecting and analyzing 198 stent ends, and for this first assumption of the potential benefits of the proposed therapy, one could consider up to 56 days a short indwelling time. It asks for next steps, which will be a study comprising longer periods to validate the treatment.

Overall, the results observed reveal a significant decrease in global encrustation in the intervention group even in the short period of time applied in this study. We also observe a higher urinary pH decrease in the experimental group, being lower urinary pH a protective factor against encrustation. To our knowledge this is the first report of a potential oral treatment to prevent double J ureteral stent encrustation by changing the urine composition of the patients.

## Conclusion

The use of an oral composition in patients indwelling a double J ureteral stent resulted in fewer stent encrustations.

## Data Availability

The data analysed during the current study are available from the corresponding author on reasonable request.

## Abbreviations

GCP: Good clinical practice
COM: Calcium oxalate monohydrate
COD: Calcium oxalate dihydrate
ER: Emergency room
